# A Comparative Genotoxicity Study of a Supraphysiological Dose of Triiodothyronine (T_3_) in Obese Rats Subjected to Either Calorie-Restricted Diet or Hyperthyroidism

**DOI:** 10.1371/journal.pone.0056913

**Published:** 2013-02-28

**Authors:** Maria Teresa De Sibio, Renata Azevedo Melo Luvizotto, Regiane Marques Castro Olimpio, Camila Renata Corrêa, Juliana Marino, Miriane de Oliveira, Sandro José Conde, Ana Lúcia dos Anjos Ferreira, Carlos Roberto Padovani, Célia Regina Nogueira

**Affiliations:** 1 Department of Internal Medicine, Botucatu Medical School - University of Sao Paulo State (UNESP), Botucatu, SP, Brazil; 2 Department of Pathology, Botucatu Medical School - University of Sao Paulo State (UNESP), Botucatu, SP, Brazil; 3 Department of Biostatistics, Biosciences Institute - University of Sao Paulo State (UNESP), Botucatu, SP, Brazil; University of Cordoba, Spain

## Abstract

This study was designed to determine the genotoxicity of a supraphysiological dose of triiodothyronine (T_3_) in both obese and calorie-restricted obese animals. Fifty male Wistar rats were randomly assigned to one of the two following groups: control (C; n = 10) and obese (OB; n = 40). The C group received standard food, whereas the OB group was fed a hypercaloric diet for 20 weeks. After this period, half of the OB animals (n = 20) were subjected to a 25%-calorie restriction of standard diet for 8 weeks forming thus a new group (OR), whereas the remaining OB animals were kept on the initial hypercaloric diet. During the following two weeks, 10 OR animals continued on the calorie restriction diet, whereas the remaining 10 rats of this group formed a new group (ORS) given a supraphysiological dose of T_3_ (25 µg/100 g body weight) along with the calorie restriction diet. Similarly, the remaining OB animals were divided into two groups, one that continued on the hypercaloric diet (OB, n = 10), and one that received the supraphysiological dose of T_3_ (25 µg/100 g body weight) along with the hypercaloric diet (OS, n = 10) for two weeks. The OB group showed weight gain, increased adiposity, insulin resistance, increased leptin levels and genotoxicity; T_3_ administration in OS animals led to an increase in genotoxicity and oxidative stress when compared with the OB group. The OR group showed weight loss and normalized levels of adiposity, insulin resistance, serum leptin and genotoxicity, thus having features similar to those of the C group. On the other hand, the ORS group, compared to OR animals, showed higher genotoxicity. Our results indicate that regardless of diet, a supraphysiological dose of T_3_ causes genotoxicity and potentiates oxidative stress.

## Introduction

Obesity is a chronic metabolic disease, which is considered a public health problem that may lead to insulin resistance and increased serum leptin levels, affecting both developed and emerging countries [1]. It may be caused by several different factors, especially the increased availability and consumption of highly palatable diets as well as low energy expenditure [2].

Obesity causes an increase in the production of reactive oxygen species (ROS) [3], leading to redox system imbalance and consequently an oxidative stress state. Such a state causes damage to lipids, proteins and especially DNA [4]. The literature thus presents several strategic approaches to deal with obese individuals, including food restriction and physical exercise [5]. The administration of thyroid hormones, alone or in combination with a hypocaloric diet, occurs illicitly despite the fact that it is not accepted as an obesity treatment by federal health agencies. Thyroid hormones are involved in the regulation of energy expenditure and thermogenesis. Thyroid dysfunction is often associated with changes in appetite and body weight as well as a decrease in both insulin resistance and serum leptin concentration [6]. However, it is not known yet if supraphysiological doses of thyroid hormones together with either obesity or a calorie restriction diet might increase DNA damage, oxidative stress, insulin resistance and serum leptin concentration. The objective of our study was to determine the effects of a supraphysiological dose of T_3_ on both obese and calorie-restricted obese rats by assessing their DNA damage and oxidative stress.

## Materials and Methods

### Animals and experimental protocol

**Figure 1:**
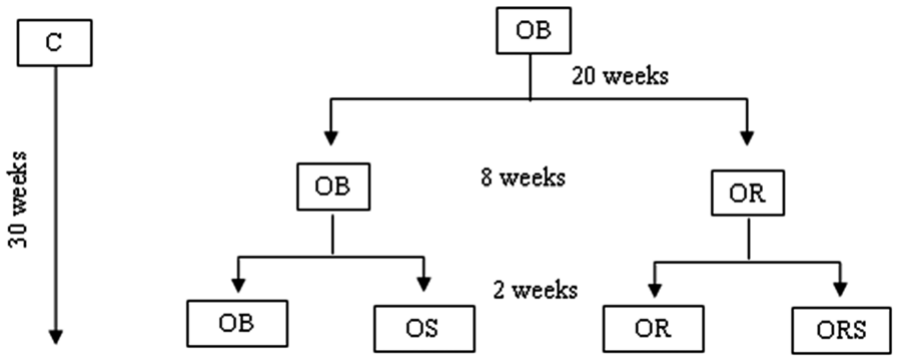
Experimental protocol. Control (C, n = 10); obese (OB, n = 50), obese given T_3_ at 25 µg/100 g BW (OS, n = 10), calorie-restricted obese (OR, n = 10) and calorie-restricted obese given T_3_ at 25 µg/100 g BW (ORS, n = 10).

Sixty-five male Wistar rats (aged 60 days and weighing approx. 150 g) were assigned for this experiment, and they were supplied by the animal facility of the Experimental Laboratory for Internal Medicine at the “Júlio de Mesquita Filho” Universidade Estadual Paulista (UNESP), Botucatu, São Paulo, Brazil. In accordance with our selection criteria [7], 15 animals excluded from the experiments. Animals were housed in individual cages under controlled room temperature (22–26°C) and lighting (12 h light/dark cycle). Animals were initially divided into two groups: control animals (C, n = 10) receiving standard food (RC Focus 1765, Agroceres®, Rio Claro, São Paulo, Brazil) containing 12.3% calories from fat and 2.95 kcal/g, and obese animals (OB, n = 40) fed a hypercaloric diet (RC Focus 2413, Agroceres®, Rio Claro, São Paulo, Brazil) containing 49.2% calories from fat and 3.65 kcal/g, as previously described [7] for 20 weeks to induce obesity. The composition of the diets is given in Table 1 [7]. After this period, 20 OB animals continued on the hypercaloric diet, whereas the other 20 formed a new group (OR) that was submitted to calorie restriction. The group then underwent calorie restriction, wherein it was placed on a normocaloric diet with 75% of the quantity ingested by the control group [8], for 8 weeks. Thus, the restriction applied was 25% of the control group. Afterwards, ten OB and ten OR animals received a supraphysiological dose of T_3_ (25 µg/100 g animal body weight) [9] for 2 weeks, as shown in Figure 1. On week 30, rats were killed by decapitation, and blood was collected and kept at -80 C for further analysis. The Botucatu Medical School Commission for Ethics in Animal Experimentation at UNESP approved the study designated experimental protocol No. 648.

**Table 1:** The calorie composition of the experimental diets.

Components	Diets	
	Normal	High-fat
Protein (%)	22	20
Carbohydrate (%)	43	26
Fat (%)	4	20
Others (%)*	31	34
% calories from protein	30	22
% calories from carbohydrate	58	29
% calories from fat	12	49
% calories from saturated fat	2.5	9.9
% calories from unsaturated fat	9.5	39.1
Calories (Kcal/g)	2.95	3.65
Fatty acid composition (%)		
Palmitic (C16∶0)	16.56	15.09
Stearic (C18∶0)	3.90	4.39
Oleic (C18∶1n9c)	27.96	37.94
Linoleic (C18∶2n6)	47.10	40.83
Others**	4.48	1.75

* Vitamins, minerals, cinders and water.

** Lauric (C12∶0), miristic (C14∶0), palmitoleic (C16∶1), Linolenic (C:18∶3n3).

### Body weight, calorie intake, metabolic efficiency

Food intake was measured daily and body weight was recorded weekly. Calorie intake was assessed by average weekly food intake multiplied by calorie value of each diet. Metabolic efficiency (ME) was calculated to analyze an animal's capacity to convert consumed food energy into body weight; this calculation used the animal's weight gain divided by the total energy ingested (kilocalories), multiplied by 100 [7].

### Body fat deposits

Total body fat was measured as the sum of epididymal, retroperitoneal, and visceral fat deposits [10]. The adiposity index was calculated by total body fat divided by final body weight multiplied by 100 [7]. This data point was utilized to confirm obesity in the animals.

### Insulin tolerance test

The insulin tolerance test (ITT) was performed to assess insulin resistance. At the end of experiment, the animals were fasted for 4 to 6 h and insulin (1 U/kg - Novolin R) was administered by intraperitoneal injection. Blood glucose levels were measured in tail by a glucometer (Accu-Chek Go Kit; Roche Dianosticas Brazil Ltda, SP, Brazil), at baseline (before administration of insulin) and 5, 10, 15, 20, 25 and 30 min after insulin administration [11].

### Serum analysis

Animals were fasted for 12–15 h, anesthetized with 50 mg/kg sodium pentobarbital i.p. and killed by decapitation. Blood was collected in dry tubes and then centrifuged at 3,000 rpm for 10 min for serum separation. Glucose levels were assessed using specific kit (CELM®, São Paulo, Brazil) for an automated colorimetric enzyme method (Technicon, RA-XT™ System, Global Medical Instrumentation, MN, USA). Serum concentrations of insulin, leptin, free T_3_, free T_4_ and thyroid stimulating hormone (TSH) were measured by immunoassay, using a microplate reader (Spectra Max 190 – Molecular Devices, Sunnyvale, CA, USA). Commercial kits were used to measure leptin, insulin (ELISA kit, Linco Research) and thyroid hormones (ELISA kit, USCN Life Science & Technology Company).

To assess lipid peroxidation, plasma malondialdehyde (MDA) was measured by high performance liquid chromatography (HPLC) with fluorometric detection [12], and the data were expressed in µM/L. Briefly, either plasma or plasma incubated with 5 mM AMVN at 42°C for 2 h was treated with BHT (5% in EtOH) followed by TCA (10% w/v) for protein precipitation. The mixture was reacted with TBA (0.4% w/v, in acetate buffer, pH 3.5) and analyzed for MDA-TBA adducts using an HPLC system with a Pecosphere-3 C18 column (83×4.6 mm) equipped with a fluorescence detector (Waters 2475 multi λ), which was set at Ex 515 nm and Em 553 nm. The HPLC mobile phase was 20 mM potassium phosphate buffer∶acetonitrile (80∶20, by volume), and the flow rate was set at 0.8 ml/min. The lower limit of detection was 0.2 pmol for the MDA-TBA adduct [12].

### Lymphocyte isolation

Lymphocytes were isolated on Ficoll–Paque gradients. Samples of peripheral blood (2 ml) were homogenized with 2 ml of phosphate-buffered saline (PBS), layered over 3 ml of Ficoll and centrifuged at 3,000 rpm for 30 min at 4°C. The lymphocyte layer was transferred to 4 ml PBS, followed by centrifugation at 2,500 rpm for 15 min, and the cell pellet was resuspended in PBS. Lymphocytes were used for the comet assay.

### DNA damage

The protocol used followed the general procedures used by Singh et al. [13] and Tice et al. [14], with some modifications. Every step was carried out under indirect light. Slides were coded and blindly analyzed. A 10-µl aliquot of peripheral blood lymphocytes was added to 120 µl of 0.5% low melting point agarose at 37°C, and the mixture was placed on slides that had been previously covered with 1.5% regular agarose, which were then coverslipped. After the agarose solidified in the refrigerator, cover slips were removed and slides immersed in lysis solution (2.5 M NaCl, 100 mM EDTA, 10 mM Tris–HCl buffer, pH 10, 1% sodium sarcosinate with 1% Triton X-100, and 10% DMSO) for 1 h. Prior to electrophoresis, slides were washed in PBS, placed in alkaline solution (pH>13) for 20 min, and then subjected to electrophoresis for 20 min at 25 V/cm and 300 mA. After electrophoresis, slides were neutralized with 0.4 M Tris–HCl buffer (pH 7.5), fixed in absolute ethanol and stored for later analysis. Positive controls (blood and spleen cells) were treated separately with 100 µM H_2_O_2_ for 5 min, in triplicate, to ensure the assay sensitivity and reproducibility. All steps were performed under dim light conditions. Slides were stained with ethidium bromide (10 mg/ml). Fifty nucleoids were assessed per slide at 400× magnification using an immunofluorescence microscope connected to an image analysis system (Comet Assay II, Perceptive Instruments, Haverhill, Suffolk, UK). “Tail intensity” (% of migrated DNA) and tail moment [the product of tail length (DNA migration) and fraction of DNA in the comet tail, i.e., % DNA in the tail] [26], were used to assess DNA damage. Since all groups showed statistical differences regarding these parameters, we decided to use “tail moment” to present our results.

### Statistical analysis

Statistical analysis was performed using the two-way analysis of variance (ANOVA) followed by Bonferroni's test with the significance level set at 5%. Data are expressed as mean ± standard deviation.

## Results

### Body weight, energy intake and metabolic efficiency

**Figure 2:**
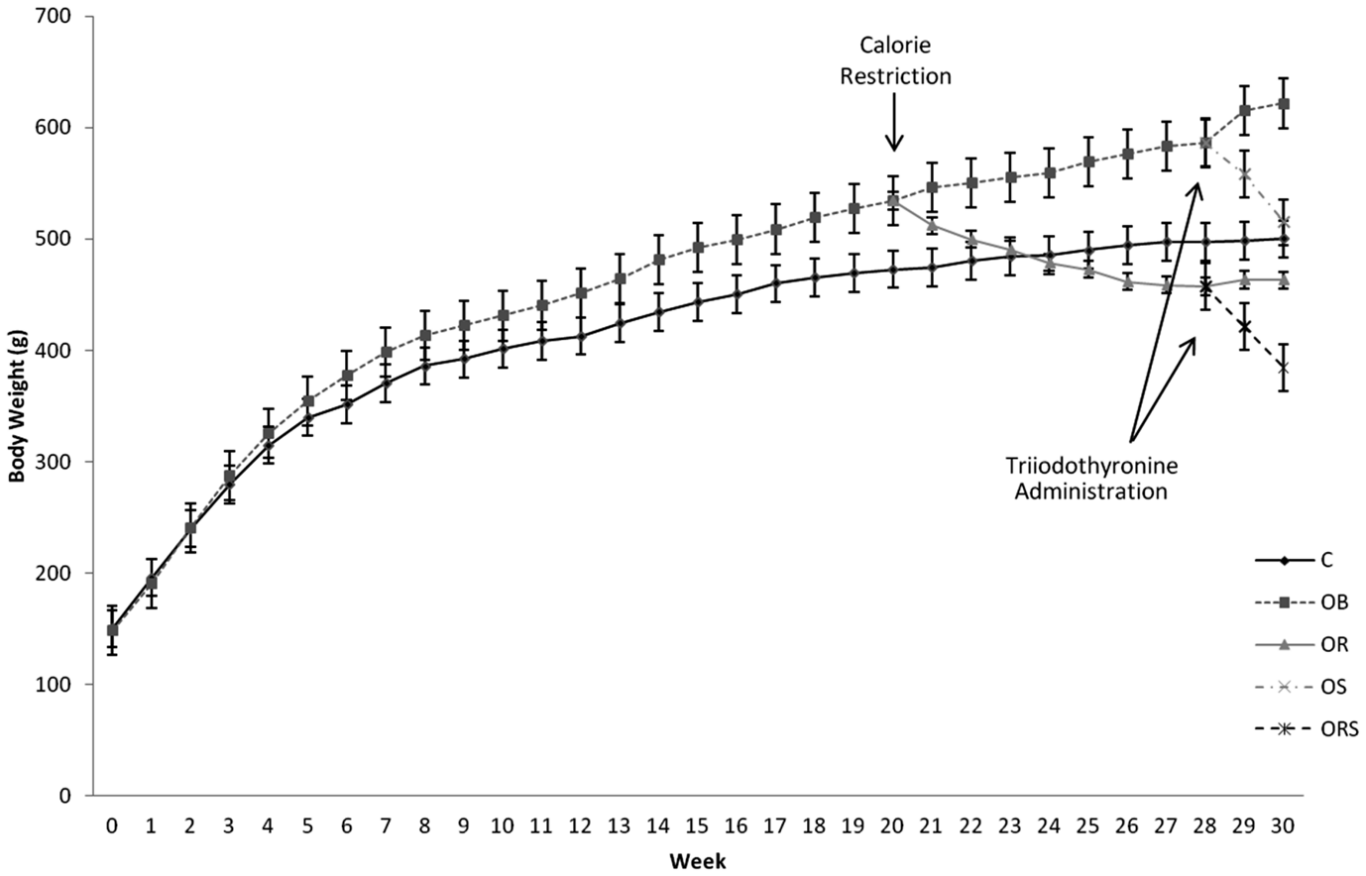
Body weight development, in grams, during a 30-week experimental period. C, control; OB, obese; OR, calorie-restricted obese; OS: obese given T_3_ at 25 µg/100 g BW; ORS: calorie-restricted obese rats given T_3_ at 25 µg/100 g BW. Data are expressed as means and 95% confidence interval.

All animals showed similar weight at the beginning of the study. After obesity induction, the animals on hypercaloric diet showed higher body weight compared to those on control diet. Food-restricted animals showed body weight loss. T_3_ treatment reduced body weight in both obese and calorie-restricted obese animals (Figure 2).

Despite the fact that the OB group ingested less food, calorie intake was higher than in the C group. The OR group showed a decrease in food ingestion and calorie intake when compared to the C and OB groups; the same occurred with the ORS group in comparison to the OS group. Comparing the OB group to the other groups, its metabolic efficiency was higher, whereas the administration of a supraphysiological dose of T_3_ reduced metabolic efficiency by reducing body weight (Table 2).

**Table 2:** Nutritional information: food intake (g), calorie intake (kcal) and metabolic efficiency (ME).

Groups	Variables		
	Intake (g/animal/day)	Intake (kcal/animal/day)	ME (%)
C×OB	25,68±1,01×22,24±2,47*	73,66±2,89×84,14±9,33**	4,57±0,27×5,64±0,51**
C×OR	25,68±1,01×20,34±057**	73,66±2,89×68,09±2,15	4,57±0,27×4,58±0,39
OB×OR	22,24±2,47× 20,34±057*	84,14±9,33×68,09±2,15**	5,64±0,51×4,58±0,39**
OB×OS	22,24±2,47× 21,43±1,23	84,14±9,33×81,09±4,67	5,64±0,51×4,56±0,40**
OR×ORS	20,34±057× 19,74±0,79	68,09±2,15×65,81±2,99	4,58±0,39×3,35±0,50**
OS×ORS	21,43±1,23×19,74±0,79	81,09±4,67×65,81±2,99**	4,56±0,40×3,35±0,50**

ME, metabolic efficiency; C, control; OB, obese; OR, calorie-restricted obese; OS: obese with 25 µg T_3_/100 g BW; ORS: calorie-restricted obese rats with 25 µg T_3_/100 g BW. Data expressed as mean ± standard deviation. ANOVA was utilized, complemented by Bonferroni's test.

* = p<0.05 and

** = p<0.01.

### Body fat deposition

The hypercaloric diet increased all fat deposits, and calorie restriction was efficient in restoring them to the C levels. Administration of T_3_ decreased retroperitoneal, visceral, and epididymal fat deposits. The OB group showed a significant increase in adiposity index when compared to C, OR, and OS. Adiposity index of OR was similar to C, and ORS group showed significant decrease when compared to OR (Table 3).

**Table 3:** Composition of body fat.

Groups	Variables			
	Epid. Fat (g)	Retro. Fat (g)	Visc. Fat (g)	Adipos. I. (%)
C×OB	8.26±1.94×13.67±2.47*	8.83±2.43×22.81±5.78*	5.95±1.52×13.16±4.08*	4.72±1.04×7.83±1.03*
C×OR	8.26±1.94×6.17±1.15	8.83±2.43×6.04±2.29	5.95±1.52×4.35±1.08	4.72±1.04×3.58±0.90
OB×OR	13.67±2.47×6.17±1.15*	22.81±5.78×6.04±2.29**	13.16±4.08×4.35±1.08*	7.83±1.03×3.58±0.90*
OB×OS	13.67±2.47×6.43±1.58*	22.81±5.78×7.98±3.89*	13.16±4.08×5.36±1.71*	7.83±1.03×3.81±1.18*
OR×ORS	6.17±1.15×1.04±0.61**	6.04±2.29×0.58±0.88**	4.35±1.08×1.06±0.60*	3.58±0.90×0.01±0.004**
OS×ORS	6.43±1.58×1.04±0.61**	7.98±3.89×0.58±0.88**	5.36±1.71×1.06±0.60*	3.81±1.18×0.01±0.004*

Epid. Fat: epididymal fat; Retro. Fat: retroperitoneal fat; Visc. Fat: visceral fat; total Fat: total body fat; Adipos. I: adiposity index; C. control; OB. obese; OR. calorie-restricted obese; OS: obese with 25 µg T_3_/100 g BW; ORS: calorie-restricted obese rats with 25 µg T_3_/100 g BW. Data expressed as mean ± standard deviation. ANOVA was utilized, complemented by Bonferroni's test.

* = p<0.05 and

** = p<0.01.

### Insulin tolerance test

**Figure 3:**
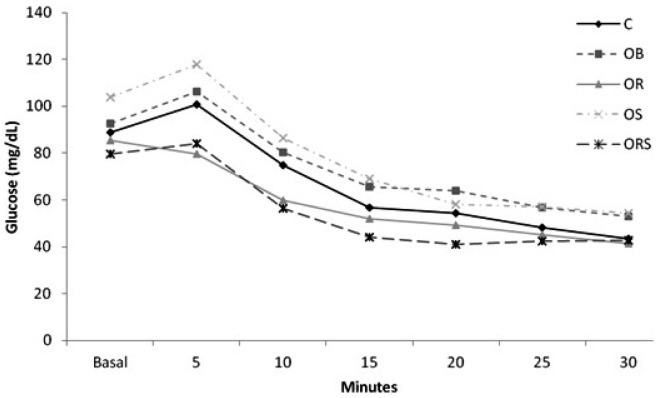
Glycemia during intraperitoneal insulin tolerance test. C, control; OB, obese; OR, calorie-restricted obese; OS: obese given T_3_ at 25 µg/100 g BW; ORS: calorie-restricted obese rats given T_3_ at 25 µg/100 g BW. Blood glucose levels were measured at baseline (before administration of insulin) and 5, 10, 15, 20, 25 and 30 min after insulin administration.

At the end of the experiments, the animals were subjected to an insulin tolerance test (Figure 3). The area under the glucose curve was determined to demonstrate insulin resistance. The OB animals exhibited an increase in insulin resistance compared to the C and OR groups. The area under the glucose curve was significantly increased in the OS group when compared to ORS (Table 4).

**Table 4:** Serum insulin, serum glucose and area under the glucose curve.

Groups	Variables		
	Insulin	Glicose	Area under the glucose curve
C×OB	1,12±0,6×1,86±0,67*	89±6,23×92±8,36*	2004±305×2229±188*
C×OR	1,12±0,6×±1,15±0,41	89±6,23×85,5±5,73	2004±305×1749±255
OB×OR	1,86±0,67×1,15±0,41*	92±8,36×85,5±5,73*	2229±188×1749±255*
OB×OS	1,86±0,67×1,14±0,33*	92±8,36×103,9±11,21	2229±188×2339±381
OR×ORS	1,15±0,41×0,53±0,23*	85,5±5,73×79,6±14,81	1749±255×1646±226
OS×ORS	1,14±0,33×0,53±0,23*	103,9±11,21×79,6±14,81	2339±381×1646±226*

C, control; OB, obese; OR, calorie-restricted obese; OS: obese with 25 µg T_3_/100 g BW; ORS: calorie-restricted obese rats with 25 µg T_3_/100 g BW. Data expressed as mean ± standard deviation. ANOVA was utilized, complemented by Bonferroni's test.

* = p<0.05 and

** = p<0.01.

### Serum measurement

Free T_3_ levels were lower in OR animals when compared with C animals, but were similar when compared with the OB group. On the other hand, free T_4_ levels were lower in OR animals when compared with OB and C animals. The T_3_ dose administered increased hormone levels in the ORS and OS animals. The OS group showed lower free T_4_ levels in comparison with the OB animals. TSH levels displayed the same behavior as free T_4_. Leptin levels increased in animals on hypercaloric diets, but decreased in the OS and ORS groups (Table 5).

**Table 5:** Free triiodothyronine; free tyroxine (free T4); Thyroid Stimulating Hormone (TSH); and leptin serum concentrations.

Groups	Variables			
	Free T3 (pmol/L)	Free T4 (pmol/L)	TSH (pmol/L)	Leptin (pmol/L)
C×OB	0.15±0.09×0.12±0.03	46.2±1.6×45.6±1.9**	14.2±0.9×12.7±1.3	3.9±0.9×9.2±1.9*
C×OR	0.15±0.09×0.08±0.02*	46.2±1.6×37.1±1.7**	14.2±0.9×10.1±1.9*	3.9±0.9×3.8±0.6
OB×OR	0.12±0.03×0.08±0.02	45.6±1.9×37.1±1.7**	12.7±1.3×10.1±1.9*	9.2±1.9×3.8±0.6*
OB×OS	0.12±0.03×0.22±0.4**	45.6±1.9×37.7±1.5**	12.7±1.3×10.9±1.2*	9.2±1.9×2.5±0.7*
OR×ORS	0.08±0.02×0.22±0.4**	37.1±1.7×37.83±1.6	10.1±1.9×11.1±1.0	3.8±0.6×0.17±0.06*
OS×ORS	0.22±0.4×0.22±0.4*	37.7±1.5×37.8±1.6	10.9±1.2×11.1±1.0	2.5±0.7×0.17±0.06

Free T3. free triiodothyronine; free T4. free tyroxine; TSH. Thyroid Stimulating Hormone; C. control; OB. obese; OR. calorie-restricted obese; OS: obese with 25 µg T_3_/100 g BW; ORS: calorie-restricted obese rats with 25 µg T_3_/100 g BW. Data expressed as mean ± standard deviation. ANOVA was utilized. complemented by Bonferroni's test.

* = p<0.05 and

** = p<0.01.

### Malondialdehyde and comet assays

**Figure 4:**
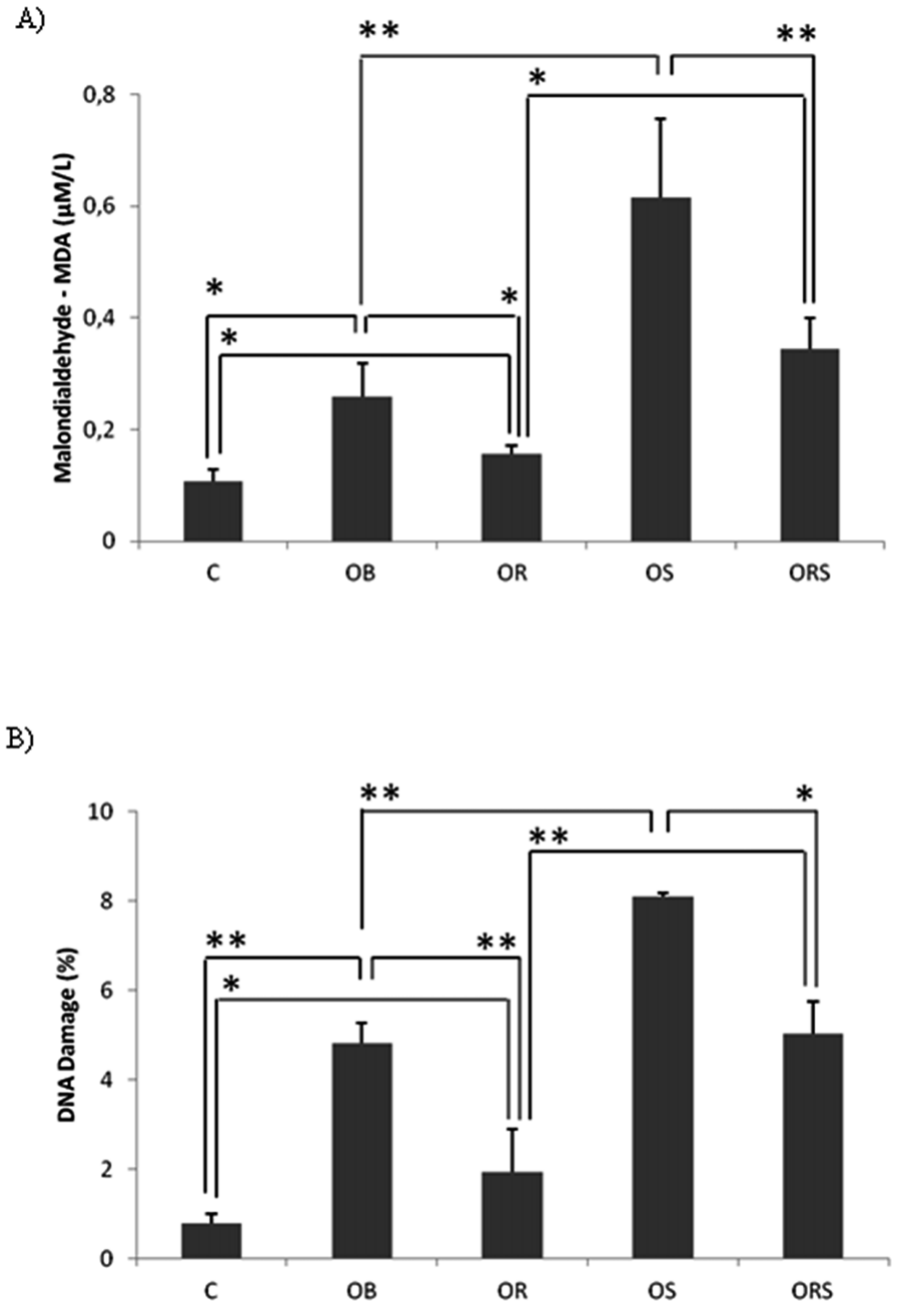
Oxidative stress analysis. A) MDA measurement. B) DNA damage evaluation. C = control; OB = obese; OR = calorie-restricted obese; OS = obese given T_3_ at 25 µg/100 g BW; ORS, calorie-restricted obese given T_3_ at 25 µg/100 g BW. Data expressed as mean ± standard deviation. ANOVA was utilized, complemented by Bonferroni's test. * = p<0.05 and ** = p<0.01.

The OB group showed higher MDA production when compared with the C and OR groups, but lower lipid peroxidation levels when compared with the OS group (p<0.01). The ORS animals showed higher DNA damage levels compared with the OR group, but lower levels compared with the OS group (Figure 4A). The DNA damage showed similar results (Figure 4B).

## Discussion

The administration of a supraphysiological dose of T_3_ augmented the serum concentration of T_3_ (Table 5) demonstrating the effectiveness of the treatment. Serum concentrations of free T_4_ and TSH were similar for C and OB animals and significantly lower in OR animals. No statistical difference was observed between animals subjected to calorie restriction and hormone administration, evincing axis suppression in restricted animals. This may reveal an adaptation of the organism to save energy, since T_3_ is a metabolically active hormone that leads to reduced body fat by increasing thermogenesis and lipid oxidation. There was a significant decrease in free T_4_ and TSH levels of OS animals when compared to OB animals, since the exogenous administration of T_3_ suppresses the endogenous secretion of TSH and the production of T_4_ by the thyroid gland [15].

As expected, OB animals gained more weight and adiposity, when compared with the C group [16], [17], [18], and the 25% restriction in calorie intake was efficient in reducing weight and adiposity in the animals [19], [20]. A supraphysiological dose of T_3_ in OB and OR animals reduced body weight and adiposity. These results were expected since clinical and experimental studies have shown that hyperthyroidism leads to increased basal metabolism [21], [22] based on the notion that triiodothyronine (T_3_) controls metabolic and energy homeostasis and influences body weight, thermogenesis, lipolysis, and metabolism of cholesterol [23]. On the other hand, TH administration did not affect the diet and calorie intake in high-fat fed (OB×OS) or calorie-restricted (OR×ORS) rats. In agreement with other studies [24], [25], metabolic efficiency was higher in the OB group. The OS group showed an increased ME compared to the ORS group, albeit lower than in OB animals, confirming the role of thyroid hormone in energy balance regulation [26].

As reported in several studies [17], [27], [28], [29], the OB animals exhibited hyperleptinemia. Caloric restriction lowered leptin levels to the same values observed in the C group [30], [31], [32], [33], and T_3_ at a dose of 25 µg/100 g BW significantly diminished leptin levels in the group treated. Despite some data show that T_3_ may directly affect leptin levels [32], [34], [35], [36], [37]. The decreased leptin levels in ORS group are better explained by decreased body fat [32], [36], [37].

Insulin resistance was evident in high-fat diet-fed rats (OB *vs* C and OR groups and OS *vs* ORS group), reinforcing the correlation between high-fat diet and insulin resistance [38], [39]. It is well established that excess thyroid hormones augment plasma glucose levels [40]. The administration of T_3_ produced a significant decrease in serum insulin levels in treated animals (Table 4). Fukuchi et al [41] suggest that high doses of thyroid hormones may be deleterious to pancreatic β-cell function, thereby reducing insulin secretion. Despite a supraphysiological dose of T_3_ (25 µg/100 g BW) decrease serum insulin levels, it did not alter insulin resistance in comparison with untreated animals (OB or OR). Nevertheless, the exact influence of thyroid hormones on insulin sensitivity and glucose metabolism remains controversial [42].

The OB animals presented greater MDA production, being in agreement with previous studies that have shown excessive fat intake may be correlated with increased markers of lipid oxidation, as MDA [43], [44]. The MDA levels were reduced by food-restriction. The possible mechanism by which calorie restriction diminishes oxidative stress is likely due to a reduction in cellular oxygen free radical production [45], and it would be correlated with lower levels of and lipid peroxidation products in the blood. Besides, it is known that calorie restriction diet improves many parameters involved in immune responses and antioxidant enzyme activities [46], [47]. Animals receiving a supraphysiological dose of T_3_, OS and ORS, showed a significant increase in MDA when compared to their respective controls (Fig. 3A). Physiological concentrations of TH do not cause increased MDA levels, however, excess concentrations of TH may lead to DNA damage and MDA production [48], [49]. In addition, experimental studies have demonstrated that animals with induced thyrotoxicosis show more lipid peroxidation, as evidenced by increased MDA levels in myocardium [22]. Since hyperthyroidism is associated with an increased metabolic rate due to an increased rate of oxygen consumption in target tissues [50], oxidative stress is regarded as a pathogenic factor in hyperthyroidism, which enhances ROS generation and produces changes in various tissue antioxidant systems, which participate in the development of hyperthyroidism-induced tissue damage [51].

Several studies indicate the use of lymphocytes to evaluate DNA oxidative damage, considering that these are excellent markers of body health conditions [52]. The comet assay affords the opportunity for correlating DNA damage and T_3_ doses [53], [54]. Although there is not much data regarding the effect of the thyroid state on protein and DNA oxidation, it does seem that hyperthyroidism promotes protein oxidation in rat liver [55]. Therefore, using lymphocytes to analyze DNA lesions in obese and food-restricted obese animals treated or not with a supraphysiological dose of TH, was found that a hypercaloric diet caused a significant increase in DNA damage (Figure 4B) [56], [57], and food restriction reduced this damage [58]. Supraphisiological dose of T_3_ had a significant increase in DNA damage, in both OS and ORS groups, when compared to their respective controls. In agreement, a cell culture study demonstrated that T_3_ augments metabolic rate and oxygen intake, generating oxidative stress, consequently leading to DNA damage [52].

Since elevated levels of thyroid hormones, including T_3_, in obesity increase resting energy expenditure avoiding fat accumulation, one may speculate whether the administration of additional thyroid hormone can be used in pharmacological treatment of obesity. However, there is no indication for thyroid hormone drugs to control body weight due to the undesirable effects, such as tachycardia, arrhythmias, fatigue, irritability, loss of bone mass, and muscle wasting, usually accompanying thyroid hormone excess [59]. Thus, the use of thyroid hormones is unsafe and harmful in the treatment of obesity, and as demonstrated in this study it leads to both increased oxidative stress and genotoxicity. The manipulation of the thyroid hormone pathway, however, seems promising. This report provides ideas for future studies on synthetic analogues of thyroid hormone, which predominantly exert effects on plasma lipids among other beneficial actions.

In summary, a hypercaloric diet in rats caused obesity, increased oxidative stress and DNA damage, whereas food restriction reduced weight, adiposity, oxidative stress and DNA damage. Supraphysiological dose of T_3_, despite decreasing body fat, increased DNA damage and oxidative stress in both obese and calorie-restricted animals, indicating that the use of supraphysiological doses of T_3_ is unsafe and harmful in obesity treatment, even combined with calorie restriction.

## References

[1] O'Brien PE, Dixon JB (2002) The extent of the problem of obesity. Am J Surg 184: 4S–8S. 12527343 10.1016/s0002-9610(02)01172-8

[2] Astrup A, Buemann B, Western P, Toubro S, Raben A, et al (1994) Obesity as an adaptation to a high-fat diet: evidence from a cross-sectional study. Am J Clin Nutr 59: 350–355. 7993398 10.1093/ajcn/59.2.350

[3] Lopez-Torres M, Gredilla R, Sanz A, Barja G (2002) Influence of aging and long-term caloric restriction on oxygen radical generation and oxidative DNA damage in rat liver mitochondria. Free Radic Biol Med 32: 882–889. 11978489 10.1016/s0891-5849(02)00773-6

[4] Halliwell B, Gutteridge JMC (1999) Free radicals in biology and medicine. Oxford: Oxford University Press. xxxi, 936 p., [919] p. of plates p.

[5] Cowburn G, Hillsdon M, Hankey CR (1997) Obesity management by life-style strategies. Br Med Bull 53: 389–408. 9246842 10.1093/oxfordjournals.bmb.a011619

[6] Kautzky-Willer A, Ludwig C, Nowotny P, Roden A, Huemer C, et al (1999) Elevation of plasma leptin concentrations in obese hyperinsulinaemic hypothyroidism before and after treatment. Eur J Clin Invest 29: 395–403. 10354196 10.1046/j.1365-2362.1999.00470.x

[7] Nascimento AF, Luvizotto RA, Leopoldo AS, Lima-Leopoldo AP, Seiva FR, et al (2011) Long-term high-fat diet-induced obesity decreases the cardiac leptin receptor without apparent lipotoxicity. Life Sci 88: 1031–1038. 21457721 10.1016/j.lfs.2011.03.015

[8] Luvizotto RA, Sibio MT, Olimpio RM, Nascimento AF, Lima-Leopoldo AP, et al (2011) Supraphysiological triiodothyronine doses diminish leptin and adiponectin gene expression, but do not alter resistin expression in calorie restricted obese rats. Horm Metab Res 43: 452–457. 21557150 10.1055/s-0031-1277187

[9] Giannocco G, DosSantos RA, Nunes MT (2004) Thyroid hormone stimulates myoglobin gene expression in rat cardiac muscle. Mol Cell Endocrinol 226: 19–26. 15489001 10.1016/j.mce.2004.07.007

[10] Levin BE, Dunn-Meynell AA (2002) Reduced central leptin sensitivity in rats with diet-induced obesity. Am J Physiol Regul Integr Comp Physiol 283: R941–948. 12228064 10.1152/ajpregu.00245.2002

[11] Claret M, Corominola H, Canals I, Nadal B, Chavanieu A, et al (2004) S 23521 decreases food intake and body weight gain in diet-induced obese rats. Obes Res 12: 1596–1603. 15536223 10.1038/oby.2004.199

[12] Li L, Duker JS, Yoshida Y, Niki E, Rasmussen H, et al (2009) Oxidative stress and antioxidant status in older adults with early cataract. Eye (Lond) 23: 1464–1468. 18806766 10.1038/eye.2008.281 PMC2695503

[13] Singh NP, McCoy MT, Tice RR, Schneider EL (1988) A simple technique for quantitation of low levels of DNA damage in individual cells. Exp Cell Res 175: 184–191. 3345800 10.1016/0014-4827(88)90265-0

[14] Tice RR, Andrews PW, Hirai O, Singh NP (1991) The single cell gel (SCG) assay: an electrophoretic technique for the detection of DNA damage in individual cells. Adv Exp Med Biol 283: 157–164. 2068983 10.1007/978-1-4684-5877-0_17

[15] Abel ED, Moura EG, Ahima RS, Campos-Barros A, Pazos-Moura CC, et al (2003) Dominant inhibition of thyroid hormone action selectively in the pituitary of thyroid hormone receptor-beta null mice abolishes the regulation of thyrotropin by thyroid hormone. Mol Endocrinol 17: 1767–1776. 12819298 10.1210/me.2003-0109

[16] Harris RB (1994) Factors influencing energy intake of rats fed either a high-fat or a fat mimetic diet. Int J Obes Relat Metab Disord 18: 632–640. 7812418

[17] Ainslie DA, Proietto J, Fam BC, Thorburn AW (2000) Short-term, high-fat diets lower circulating leptin concentrations in rats. Am J Clin Nutr 71: 438–442. 10648255 10.1093/ajcn/71.2.438

[18] Roberts CK, Vaziri ND, Barnard RJ (2002) Effect of diet and exercise intervention on blood pressure, insulin, oxidative stress, and nitric oxide availability. Circulation 106: 2530–2532. 12427646 10.1161/01.cir.0000040584.91836.0d

[19] Thompson WG, Rostad Holdman N, Janzow DJ, Slezak JM, Morris KL, et al (2005) Effect of energy-reduced diets high in dairy products and fiber on weight loss in obese adults. Obes Res 13: 1344–1353. 16129716 10.1038/oby.2005.163

[20] Daly ME, Paisey R, Millward BA, Eccles C, Williams K, et al (2006) Short-term effects of severe dietary carbohydrate-restriction advice in Type 2 diabetes–a randomized controlled trial. Diabet Med 23: 15–20. 16409560 10.1111/j.1464-5491.2005.01760.x

[21] Ahmed OM, Ahmed RG, El-Gareib AW, El-Bakry AM, Abd El-Tawab SM (2012) Effects of experimentally induced maternal hypothyroidism and hyperthyroidism on the development of rat offspring: II-The developmental pattern of neurons in relation to oxidative stress and antioxidant defense system. Int J Dev Neurosci 30: 517–537. 22664656 10.1016/j.ijdevneu.2012.04.005

[22] Rybus-Kalinowska B, Zwirska-Korczala K, Kalinowski M, Kukla M, Birkner E, et al (2008) Activity of antioxidative enzymes and concentration of malondialdehyde as oxidative status markers in women with newly diagnosed Graves-Basedow disease and after thiamazole therapy leading to euthyroidism. Pol Arch Med Wewn 118: 420–425. 18714737

[23] Rotondi M, Magri F, Chiovato L (2011) Thyroid and obesity: not a one-way interaction. J Clin Endocrinol Metab 96: 344–346. 21296993 10.1210/jc.2010-2515

[24] Gaiva MH, Couto RC, Oyama LM, Couto GE, Silveira VL, et al (2001) Polyunsaturated fatty acid-rich diets: effect on adipose tissue metabolism in rats. Br J Nutr 86: 371–377. 11570989 10.1079/bjn2001392

[25] Estadella D, Oyama LM, Damaso AR, Ribeiro EB, Oller Do Nascimento CM (2004) Effect of palatable hyperlipidic diet on lipid metabolism of sedentary and exercised rats. Nutrition 20: 218–224. 14962690 10.1016/j.nut.2003.10.008

[26] Vendrell J, Broch M, Vilarrasa N, Molina A, Gomez JM, et al (2004) Resistin, adiponectin, ghrelin, leptin, and proinflammatory cytokines: relationships in obesity. Obes Res 12: 962–971. 15229336 10.1038/oby.2004.118

[27] Barnes MJ, Lapanowski K, Conley A, Rafols JA, Jen KL, et al (2003) High fat feeding is associated with increased blood pressure, sympathetic nerve activity and hypothalamic mu opioid receptors. Brain Res Bull 61: 511–519. 13679250 10.1016/s0361-9230(03)00188-6

[28] Bell-Anderson KS, Bryson JM (2004) Leptin as a potential treatment for obesity: progress to date. Treat Endocrinol 3: 11–18. 15743109 10.2165/00024677-200403010-00002

[29] Vasselli JR, Weindruch R, Heymsfield SB, Pi-Sunyer FX, Boozer CN, et al (2005) Intentional weight loss reduces mortality rate in a rodent model of dietary obesity. Obes Res 13: 693–702. 15897478 10.1038/oby.2005.78

[30] Elliott JC, Harrold JA, Brodin P, Enquist K, Backman A, et al (2004) Increases in melanin-concentrating hormone and MCH receptor levels in the hypothalamus of dietary-obese rats. Brain Res Mol Brain Res 128: 150–159. 15363890 10.1016/j.molbrainres.2004.06.010

[31] Levin BE, Dunn-Meynell AA (2004) Chronic exercise lowers the defended body weight gain and adiposity in diet-induced obese rats. Am J Physiol Regul Integr Comp Physiol 286: R771–778. 14695115 10.1152/ajpregu.00650.2003

[32] Wolfe BE, Jimerson DC, Orlova C, Mantzoros CS (2004) Effect of dieting on plasma leptin, soluble leptin receptor, adiponectin and resistin levels in healthy volunteers. Clin Endocrinol (Oxf) 61: 332–338. 15355449 10.1111/j.1365-2265.2004.02101.x

[33] Viguerie N, Vidal H, Arner P, Holst C, Verdich C, et al (2005) Adipose tissue gene expression in obese subjects during low-fat and high-fat hypocaloric diets. Diabetologia 48: 123–131. 15624093 10.1007/s00125-004-1618-x

[34] Zabrocka L, Klimek J, Swierczynski J (2006) Evidence that triiodothyronine decreases rat serum leptin concentration by down-regulation of leptin gene expression in white adipose tissue. Life Sci 79: 1114–1120. 16624326 10.1016/j.lfs.2006.03.009

[35] Pinkney JH, Goodrick SJ, Katz J, Johnson AB, Lightman SL, et al (1998) Leptin and the pituitary-thyroid axis: a comparative study in lean, obese, hypothyroid and hyperthyroid subjects. Clin Endocrinol (Oxf) 49: 583–588. 10197072 10.1046/j.1365-2265.1998.00573.x

[36] Schrauwen P, Westerterp KR (2000) The role of high-fat diets and physical activity in the regulation of body weight. Br J Nutr 84: 417–427. 11103212 10.1017/s0007114500001720

[37] Johnstone AM, Murison SD, Duncan JS, Rance KA, Speakman JR (2005) Factors influencing variation in basal metabolic rate include fat-free mass, fat mass, age, and circulating thyroxine but not sex, circulating leptin, or triiodothyronine. Am J Clin Nutr 82: 941–948. 16280423 10.1093/ajcn/82.5.941

[38] Brown JL, Spicer MT, Spicer LJ (2002) Effect of high-fat diet on body composition and hormone responses to glucose tolerance tests. Endocrine 19: 327–332. 12624434 10.1385/ENDO:19:3:327

[39] van Dam RM, Willett WC, Rimm EB, Stampfer MJ, Hu FB (2002) Dietary fat and meat intake in relation to risk of type 2 diabetes in men. Diabetes Care 25: 417–424. 11874924 10.2337/diacare.25.3.417

[40] Dimitriadis GD, Raptis SA (2001) Thyroid hormone excess and glucose intolerance. Exp Clin Endocrinol Diabetes 109 Suppl 2: S225–239. 11460573 10.1055/s-2001-18584

[41] Fukuchi M, Shimabukuro M, Shimajiri Y, Oshiro Y, Higa M, et al (2002) Evidence for a deficient pancreatic beta-cell response in a rat model of hyperthyroidism. Life Sci 71: 1059–1070. 12088765 10.1016/s0024-3205(02)01791-5

[42] Setia S, Sridhar MG, Koner BC, Bobby Z, Bhat V, et al (2007) Increased insulin sensitivity in intrauterine growth retarded newborns–do thyroid hormones play a role? Clin Chim Acta 376: 37–40. 16914130 10.1016/j.cca.2006.07.007

[43] Willett WC (2001) Diet and breast cancer. J Intern Med 249: 395–411. 11350564 10.1046/j.1365-2796.2001.00822.x

[44] Wynder EL, Cohen LA, Muscat JE, Winters B, Dwyer JT, et al (1997) Breast cancer: weighing the evidence for a promoting role of dietary fat. J Natl Cancer Inst 89: 766–775. 9182974 10.1093/jnci/89.11.766

[45] Mattson MP, Wan R (2005) Beneficial effects of intermittent fasting and caloric restriction on the cardiovascular and cerebrovascular systems. J Nutr Biochem 16: 129–137. 15741046 10.1016/j.jnutbio.2004.12.007

[46] Nikolich-Zugich J, Messaoudi I (2005) Mice and flies and monkeys too: caloric restriction rejuvenates the aging immune system of non-human primates. Exp Gerontol 40: 884–893. 16087306 10.1016/j.exger.2005.06.007

[47] Sohal RS, Weindruch R (1996) Oxidative stress, caloric restriction, and aging. Science 273: 59–63. 8658196 10.1126/science.273.5271.59 PMC2987625

[48] Hudig F, Bakker O, Wiersinga WM (1997) Tri-iodothyronine prevents the amiodarone-induced decrease in the expression of the liver low-density lipoprotein receptor gene. J Endocrinol 152: 413–421. 9071962 10.1677/joe.0.1520413

[49] Magsino CH Jr, Hamouda W, Ghanim H, Browne R, Aljada A, et al (2000) Effect of triiodothyronine on reactive oxygen species generation by leukocytes, indices of oxidative damage, and antioxidant reserve. Metabolism 49: 799–803. 10877210 10.1053/meta.2000.6263

[50] Videla LA (2000) Energy metabolism, thyroid calorigenesis, and oxidative stress: functional and cytotoxic consequences. Redox Rep 5: 265–275. 11145101 10.1179/135100000101535807

[51] Fernandez V, Barrientos X, Kipreos K, Valenzuela A, Videla LA (1985) Superoxide radical generation, NADPH oxidase activity, and cytochrome P-450 content of rat liver microsomal fractions in an experimental hyperthyroid state: relation to lipid peroxidation. Endocrinology 117: 496–501. 2990853 10.1210/endo-117-2-496

[52] Oldham KM, Wise SR, Chen L, Stacewicz-Sapuntzakis M, Burns J, et al (2002) A longitudinal evaluation of oxidative stress in trauma patients. JPEN J Parenter Enteral Nutr 26: 189–197. 12005461 10.1177/0148607102026003189

[53] Sanchez-Suarez P, Ostrosky-Wegman P, Gallegos-Hernandez F, Penarroja-Flores R, Toledo-Garcia J, et al (2008) DNA damage in peripheral blood lymphocytes in patients during combined chemotherapy for breast cancer. Mutat Res 640: 8–15. 18207203 10.1016/j.mrfmmm.2007.11.008

[54] Miyamae Y, Zaizen K, Ohara K, Mine Y, Sasaki YF (1998) Detection of DNA lesions induced by chemical mutagens by the single cell electrophoresis (Comet) assay. 1. Relationship between the onset of DNA damage and the characteristics of mutagens. Mutat Res 415: 229–235. 9714818 10.1016/s1383-5718(97)00192-7

[55] Venditti P, Di Meo S (2006) Thyroid hormone-induced oxidative stress. Cell Mol Life Sci 63: 414–434. 16389448 10.1007/s00018-005-5457-9 PMC11136030

[56] Parks EJ (2001) Recent findings in the study of postprandial lipemia. Curr Atheroscler Rep 3: 462–470. 11602066 10.1007/s11883-001-0036-5

[57] Hennig B, Toborek M, McClain CJ (2001) High-energy diets, fatty acids and endothelial cell function: implications for atherosclerosis. J Am Coll Nutr 20: 97–105. 11349944 10.1080/07315724.2001.10719021

[58] Heydari AR, Unnikrishnan A, Lucente LV, Richardson A (2007) Caloric restriction and genomic stability. Nucleic Acids Res 35: 7485–7496. 17942423 10.1093/nar/gkm860 PMC2190719

[59] Reinehr T (2010) Obesity and thyroid function. Mol Cell Endocrinol 316: 165–171. 19540303 10.1016/j.mce.2009.06.005

